# A Comparative Study on Simulated Chairside Grinding and Polishing of Monolithic Zirconia

**DOI:** 10.3390/ma15062202

**Published:** 2022-03-16

**Authors:** Mohit Kheur, Tabrez Lakha, Saleha Shaikh, Supriya Kheur, Batul Qamri, Lee Wan Zhen, Nadin Al-Haj Husain, Mutlu Özcan

**Affiliations:** 1 Department of Prosthodontics and Implantology, M.A. Rangoonwala College of Dental Sciences & Research Centre, Pune 411001, India; mkheur@gmail.com (M.K.); salehashaik221@gmail.com (S.S.); 2 Department of Oral Pathology and Microbiology, D.Y. Patil Dental College, D.Y. Patil Vidyapeeth, Pune 411018, India; drskheur@gmail.com; 3 Specialty Dental Care, Pune 411037, India; bqamri@gmail.com; 4 National Dental Centre Singapore, Department of Restorative Dentistry, Singapore 168938, Singapore; leewzhen@gmail.com; 5 Center of Dental Medicine, Division of Dental Biomaterials, Clinic for Reconstructive Dentistry, University of Zurich, 8032 Zurich, Switzerland; nadin.al-haj-husain@zmk.unibe.ch (N.A.-H.H.); mutluozcan@hotmail.com (M.Ö.); 6 Department of Reconstructive Dentistry Gerodontology, School of Dental Medicine, University of Bern, 3010 Bern, Switzerland

**Keywords:** flexural strength, hardness, monolithic zirconia, polishing, X-ray diffraction analysis

## Abstract

This study evaluated the effects of different simulated chairside grinding and polishing protocols on the physical and mechanical properties of surface roughness, hardness, and flexural strength of monolithic zirconia. Sintered monolithic zirconia specimens (15 mm × 3 mm × 3 mm) were abraded using three different burs: diamond bur, modified diamond bur (zirconia specified), and tungsten carbide bur, along with a group of unprepared specimens that served as a control group. The study was divided into two phases, Phase 1 and Phase 2. Surface roughness, surface hardness, and flexural strength were assessed before and after the grinding procedure to determine the ‘best test group’ in Phase 1. The best abrasive agent was selected for Phase 2 of the study. The specimens in Phase 2 underwent grinding with the best abrasive agent selected. Following the grinding, the specimens were then polished using commercially available diamond polishing paste, a porcelain polishing kit, and an indigenously developed low-temperature sintered zirconia slurry. The physical and mechanical properties were again assessed. Results were analyzed using one-way ANOVA test. Specimens were observed under scanning electron microscopy (SEM) and X-ray diffraction (XRD) for their microstructure and crystalline phases, respectively. Grinding with diamond burs did not weaken zirconia (*p* > 0.05) but produced rougher surfaces than the control group (*p* < 0.05). Tungsten carbide burs did not significantly roughen the zirconia surface. However, specimens ground by tungsten carbide burs had a significantly reduced mean flexural strength (*p* < 0.05) and SEM revealed fine surface cracks. Phase transformation was not detected by XRD. Polishing with commercially available polishing agents, however, restored the surface roughness levels to the control group. Dental monolithic zirconia ground with tungsten carbide burs had a significantly reduced flexural strength and a smooth but defective surface. However, grinding with diamond burs roughened the zirconia surface. These defects may be reduced by polishing with commercially available polishing agents. The use of tungsten carbide burs for grinding dental zirconia should not be advocated. Grinding with diamond abrasives does not weaken zirconia but requires further polishing with commercially available polishing agents.

## 1. Introduction

The increasing aesthetic demands of patients combined with advances in ceramic technology have led to the emergence and acceptance of all-ceramic restorations in dentistry. The literature has reported that the percentage of all ceramic fixed restorations increased from 23.9% to 80.2% from the year 2008 to 2016 [1].

From a clinical standpoint, there are three major ceramic groups. These include glassy materials (castable, machinable, and pressable glass infiltrated), particle-filled glasses (leucite), and polycrystalline ceramics (zirconia) [2].

Zirconia is commonly veneered with feldspathic porcelain for superior aesthetics albeit with high incidence of chipping, which led to the advent of monolithic zirconia. Due to their high fracture resistance, monolithic variants are indicated for high-stress-bearing posterior areas [3,4]. As mentioned before, the polycrystalline ceramic zirconia exists in three different phases: monoclinic (M), tetragonal (T), and cubic (C) phases. Out of these, the tetragonal phase exhibits superior physical and mechanical properties. At room temperature, stabilization of tetragonal phase zirconia is achieved by the incorporation of metal oxides (CaO, MgO, CeO2, Y_2_O_3_) to prevent the monoclinic (T→M) transformation. However, under stress, 3Y-TZP undergoes tetragonal to monoclinic (T→M) transformation. This transformation is associated with a local volumetric increase (∼4.5%), which in turn compresses the crack defects, thereby preventing further propagation, resulting in an increase in the flexural strength of zirconia [5]. However, this process may contribute to early ageing or low-temperature degradation in the presence of water, leading to surface degradation and a reduction in strength.

Sun et al. stated that zirconia crowns of 1 mm occlusal thickness have adequate fracture resistance, as compared with metal–ceramic crowns [6]. Crowns and bridges often require chairside and subsequent laboratory adjustments for the removal of occlusal interferences and/or for correction of contours prior to cementation, leaving an irregular rough surface [7]. However, there are no guidelines published in the literature that advise clinicians about the maximum permissible depth of grinding monolithic zirconia for chairside adjustments. Zucuni et al. [8], İşeri et al. [7], Mohammadi-Bassir et al. [9] reported that grinding and polishing induce microcracks, which in turn affect the strength of the restoration. Consequently, rough surfaces can eventually lead to wear of opposing natural dentition, plaque accumulation, gingival inflammation, and periodontal disease. There has been an increased interest in research on the optimum chairside grinding and finishing protocols for zirconia.

Studies by Goo et al. [10] and Park et al. [11] evaluated the surface roughness and topography of monolithic zirconia using different chairside polishing systems and concluded that the surfaces polished with zirconia polishers were smoother than those polished by a porcelain polisher. Most studies have evaluated the effect of different surface treatments, such as grinding with a fine-grit (30 µm) diamond bur without water coolant in an air-turbine handpiece, air-particle abrasion, rubber-point polishing in a contra-angle handpiece, and simulating minor surface abrasion of zirconia [12]. However, no study has been carried out in an in vitro model wherein zirconia-specific abrasives are used to grind zirconia surfaces to varying residual thicknesses in order to study their effect on the material’s physical and mechanical properties.

There have been numerous studies in the literature demonstrating effect of different grinding and polishing methods on the flexural strength of zirconia. However, no study has been conducted in an in vitro model wherein zirconia-specific abrasives are used and their effect on the physical properties is evaluated when the zirconia specimens are abraded to a greater depth. 

This study was therefore undertaken to evaluate the changes in the physical and mechanical properties of monolithic zirconia after grinding using various commercially available abrasives and after polishing using conventional polishing techniques and an indigenously developed resintering protocol.

For situations where excessive surface reduction has been carried out, an interesting method of ‘repairing’ a ground zirconia surface would be the application of a zirconia slurry on the abraded area, followed by sintering at a lower temperature. This study was therefore undertaken to evaluate the changes in the physical and mechanical properties of monolithic zirconia after grinding using various commercially available abrasives and polishing using conventional polishing techniques and an indigenously developed resintering protocol. The first null hypothesis is that different surface grinding and polishing agents would not affect the surface roughness, hardness, and flexural strength of monolithic zirconia. The second null hypothesis is that there is no statistical difference between different surface grinding and polishing procedures. 

## 2. Materials and Methods

**Figure 1:**
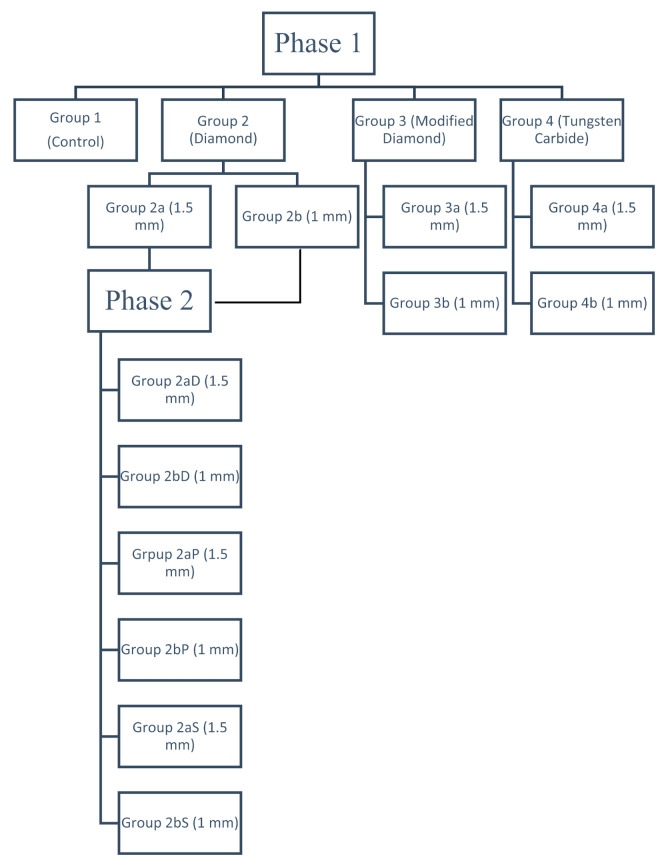
Flowchart of the experimental procedures.

The study was carried out in two phases. Phase 1 was conducted to determine the physical and mechanical properties of the ground specimens. The grinding protocol of the best test group from this phase was used in Phase 2 to determine the optimum polishing protocols (Figure 1).

### 2.1. Phase 1: Determination of Optimum Chairside Abrasion Protocols for Monolithic Zirconia

#### 2.1.1. Specimen Preparation

Sample size estimation was performed using the following method: The sample size was determined by using the effect sizes from a previously published study [13] and with the help of the following formula:

$$n\;\left(\mathrm{Per}\;\mathrm{Group}\right)=2{\left[\;\frac{\left(\;Z\alpha /2+{\;Z}_{\beta }\right)\sigma }{\Delta }\right]}^{2}$$

where n = sample size (per group); Z_α/2_ = (1.96) for 95% confidence (i.e., α = 0.05) = 1.96; Z_β_ = cut-off value for power (1 − β) = 0.8416 (80.0% power); Δ = mean difference to be detected (minimum difference) = 2.0 units of Delta E; and Δ/δ = effect size in SD units = 0.600. Thus, the sample size according to this formula is 7.5 ≅ 8 (minimum per group) (i.e., total 56 (minimum)).

**Figure 2:**
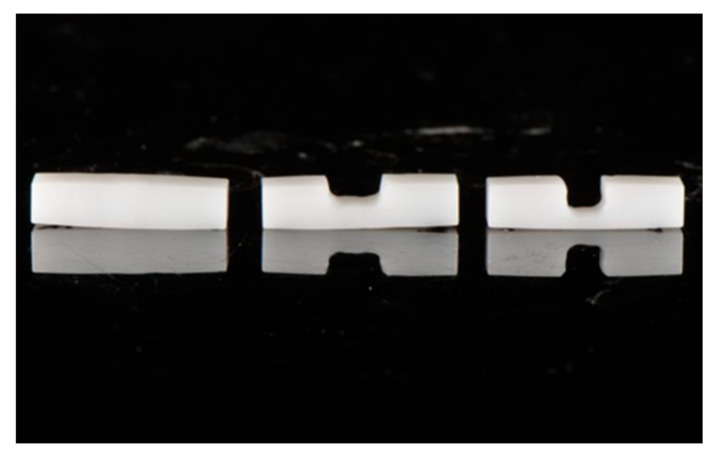
Samples abraded to 1.5 mm and 1 mm residual thickness.

**Figure 3:**
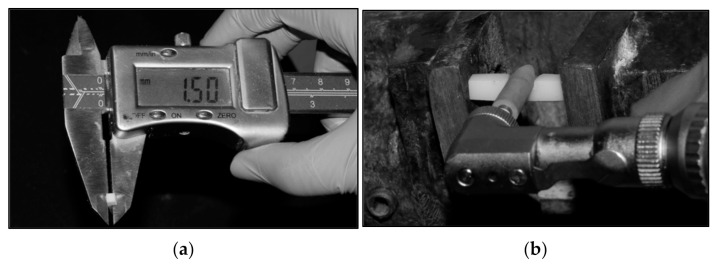
(**a**) The specimens had dimensions of 15 mm length × 3 mm width × 3 mm thickness. The length, width, and thickness were verified with a digital caliper for standardization. (**b**) Specimens polished using a porcelain polishing kit. Specimens in this group underwent polishing with the commercially available polishing kit (Shofu Porcelain Laminate). Polishing was performed with stroke movements for 30 s by a single trained operator, following the sequence (yellow followed by white polishing points) and speed recommendations (10,000–12,000 rpm) for the system.

A total of 140 presintered specimens (Figure 2) were fabricated by milling yttrium-stabilized zirconia blanks (3M™ ESPE™ Lava™, St. Paul, MN, USA) using a milling machine at the presintered stage. The prepared specimens were then sintered in a furnace at 1500 °C for 2 h. After sintering, the specimens were ultrasonically cleaned with ethyl alcohol and air-dried. The final specimens had dimensions of 15 mm length × 3 mm width × 3 mm thickness. Each specimen’s length, width, and thickness were verified with a digital caliper for standardization (Figure 3a,b). Specimens were divided into four groups with 20 units per group. 

#### 2.1.2. Grinding Procedure

Group I: control group; group II: ground using diamond burs; group III: ground using zirconia-specific diamond points; group IV: ground using tungsten carbide burs.

A customized assembly was designed to achieve controlled grinding procedures. It was used to mount both the handpiece and the specimen. The handpiece was clamped on a flat platform that could slide sideways. Another clamp to stabilize the specimen was attached to the assembly. Burs inserted in the handpiece were oriented parallel to and positioned in contact with the specimen. The area to be ground was marked with a permanent marking pen in the middle portion approximately 5 mm in length. This was done to ensure that the entire zirconia surface was ground, and the grinding process was performed until complete elimination of the marking made in the center of the specimen. Each specimen was ground with a new bur (1 bur per specimen). The specimens were abraded to a residual thickness of 1.5 mm. This methodology was previously described and employed by Pereira et al. and Sandhu et al. [14,15].

In order to simulate chairside clinical conditions, water irrigation was performed during the grinding (Table 1) and polishing (Table 2) of all the specimens as described previously by Preis et al. [16]. The samples were abraded to residual thicknesses of 1.5 and 1.0 mm, respectively, following which the specimens were ultrasonically cleaned with water and air-dried before testing.

The specimens were tested for three physical and mechanical properties, namely, surface roughness, surface hardness, and flexural strength.

#### 2.1.3. Surface Roughness

The surface roughnesses of all specimens were measured before and after abrasion using a contact profilometer (Perthometer SP6, Feinpruf Perthen, Mahr, Gottingen, Germany) and an integrated software program (MarSurfXR20, Mahr, Göttingen, Germany) using these settings: velocity: 0.1 mm/s; critical wavelength: 0.25; and transverse length: 2.4 mm. The direction of the measurement was at the right angle to the direction of the abrasion. Ra (arithmetic average roughness) was then calculated. Three repeated measurements were performed on each specimen.

#### 2.1.4. Surface Hardness

Diamond-shaped indents were induced using a Vickers hardness tester (Struers, Glasgow, Scotland) with a load of P = 9.807 N for 15 s. A micrometer screw gauge was used to measure the size of each diagonal distance within the microscope to obtain an average diagonal distance (D) in micrometers. The surface area was calculated, and the Vickers hardness (VH) was obtained using the following formula: VH = (2 P/D^2^)sin (68°) = (1.854 P/D^2^); Vickers constant: 1.854; angle of indentation: 68°. Size of the notched boxes was 5 mm × 3 mm × 3 mm. The stylus head of the contact profilometer and the Vickers indenter were accommodated. Three repeated measurements were made on each specimen.

#### 2.1.5. Flexural Strength

The flexural strength (Newton) of all specimens was measured according to International Organization for Standardization standard 6872 using the universal testing machine (model 5566; Instron Corp., Norwood, MA, USA). Once the specimens were positioned on a circular fixture, an increasing load (0.5 mm/min) was applied throughout a circular tungsten piston (Ø = 2 mm) up to failure. 

The specimens were also subjected to SEM and XRD analysis. The phase transformation of zirconia was investigated by X-ray powder diffraction technique (Bruker, D8 Advance) using CuKα (1.54) X-rays. The diffraction profiles were acquired in the 2 θ range from 20° to 80°, where θ is the angle of reflection with a step size of 0.03 and a scan rate of 0.6 s/step as performed by Sandhu et al. The relative amount of phase transformation for the specimens was determined as described by Karakoca and Yilmaz.

Surface characterization of the control, abraded, and polished zirconia groups was performed using a scanning electron microscopy device (SEM, Zeiss MERLIN Field-Emission SEM, Carl Zeiss NTS GmbH, Oberkochen, Germany). The specimens were cleansed in an ultrasonic cleaner, dried in a vacuum desiccator, and mounted on aluminum stubs for viewing under different magnifications.

The abrasive agent that showed best overall results when the surface roughness changed and the flexural strength was assessed was selected as the ‘Best Test Group’.

### 2.2. Phase 2: Determination of Optimum Chairside Polishing Protocols for Abraded Monolithic Zirconia

All the samples were abraded using the same methodology employed in Phase 1 of the study. Based on the results in Phase 1, diamond points (group 2a,b) were selected for further testing in Phase 2. All the samples in Phase 2 were abraded using diamond burs. These samples were further divided into 3 groups with 20 specimens, each abraded to residual thicknesses of 1.5 and 1.0 mm.

Three polishing protocols were followed in this study. The factors considered in selecting the said protocols were in tandem with the manufacturer’s instructions.

The polishing systems used were as follows:

Group 2aD, 2bD: specimens polished using diamond polishing paste (Signum HP diamond, Kulzer, Germany) using a polishing wheel (Z-Shine, Dental Creations Ltd., Waco, TX, USA); Group 2aP, 2bP: specimens polished using a commercially available ceramic polishing kit (Shofu Dental Corporation, San Marcos, TX, USA); Group 2aS, 2aS: specimens polished using the application of the novel zirconia slurry and a low-temperature resintering procedure.

After abrading the samples with the selected abrasive (diamond bur) as described previously for Phase 1, the specimens were ultrasonically cleaned and air-dried. The specimens were subjected to the respective polishing procedure. The same customized assembly, as described previously, was employed to achieve a controlled polishing procedure. 

Specimens in Group 2aD, 2bD underwent polishing with a rubber wheel and diamond polishing paste (2–4 µm). Polishing was performed with stroke movements for 30 s by a single trained operator following the recommended speed (10,000–12,000 rpm). Specimens in Group 2aP, 2bP underwent polishing with the commercially available polishing kit (Shofu Porcelain Laminate). Polishing was performed with stroke movements for 30 s by a single trained operator (following the color sequence) and speed recommendations (10,000–12,000 rpm) for the system [11]. The specimens of Group 2aS, 2bS underwent polishing in the same way after the application of the indigenously developed polishing slurry.

The zirconia particles used in the slurry were made by milling presintered zirconia blocks (3M^TM^ ESPE^TM^ Lava^TM^, St. Paul, MN, USA) left over from the milling of zirconia crowns or bridges. The blocks were milled using a milling machine (Planetary Mill, Pulverisette 5, Idar-Oberstein, Germany), generating fine-diameter zirconia particles. For the slurry, glazing liquid was used as lubricant in a 1:1 ratio, achieved by a mass, and mixed using a plastic spatula for 5 min. Using an applicator brush, the polishing paste was applied onto the center of the specimens, followed by low-temperature sintering (450 °C) for 7 min.

After polishing, the surface roughness R_a_, surface hardness, and flexural strength values were determined again as previously described. The results were tabulated and subjected to statistical analysis. The statistical analysis was performed between each subgroup a and b within the same group, as well as between the same subgroups of different groups. The following statistical tests were performed for each analysis: surface roughness, surface hardness, and flexural strength. As the data followed a normal parametric distribution (as per the Anderson–Darling test), ANOVA followed by ad hoc Bonferroni simultaneous tests at α = 0.05 were used. Similarly, an ANOVA test was performed to evaluate the statistical difference in flexural strength. Based on the results of the flexural strength, the group that showed least reduction of flexural strength was selected for the Phase 2 analysis. For determining the optimum method for chairside polishing, ANOVA followed by ad hoc Bonferroni simultaneous tests at α = 0.05 were used. *p*-Values less than 0.05 were considered to be statistically significant in all tests.

## 3. Results

### 3.1. Phase

In Phase 1, the ‘best test group’ was determined based on three physical and mechanical values, which were surface roughness, surface hardness, and flexural strength.

#### 3.1.1. Surface Roughness

**Figure 4:**
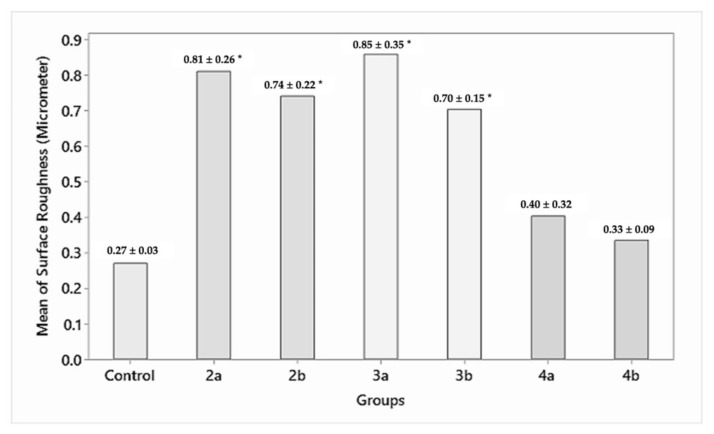
Surface roughness (Ra) in µm of the abraded zirconia samples (microns). * Statistically significant difference between the groups.

While the control group (group 1) specimens had a surface roughness of 0.27 ± 0.03 µm, the specimens in the test groups—group 2a, 2b (0.81 ± 0.26, 0.74 ± 0.22 µm), group 3a, 3b (0.85 ± 0.35, 0.70 ± 0.15 µm)—showed a statistically significant (*p* = 0.000) increase in surface roughness for 1.5 and 1 mm residual thicknesses, respectively, and group 4a, 4b (0.40 ± 0.32, 0.33 ± 0.09 µm) showed a statistically insignificant (*p* = 0.824) increase in surface roughness for 1.5 and 1 mm residual thicknesses, respectively (Figure 4).

#### 3.1.2. Surface Hardness

**Figure 5:**
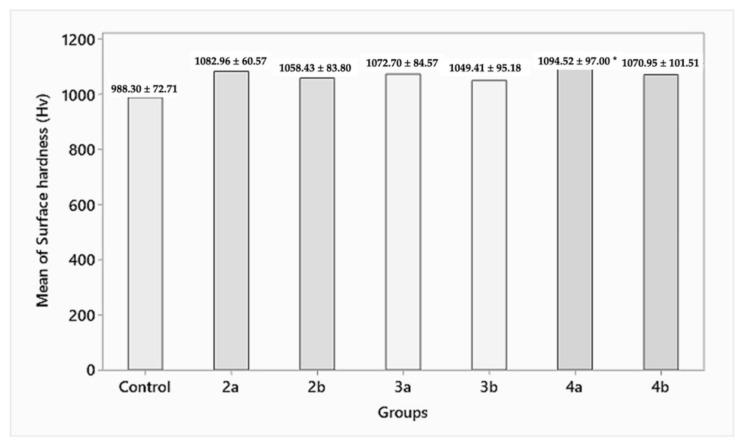
Surface hardness of the abraded zirconia samples (HV). * Statistically significant difference between the groups.

While the control group (group 1) had a surface hardness of 988.30 ± 72.71 HV, the specimens in the test groups—group 2a, 2b (1082.96 ± 60.57, 1058.43 ± 83.80 HV); group 3a, 3b (1072.70 ± 84.57, 1049.41 ± 95.18 HV); and group 4a, 4b (1094.52 ± 97.00, 1070.95 ± 101.51 HV)—showed a statistically insignificant (*p* > 0.05) increase in surface hardness for 1.5 and 1 mm residual thicknesses, respectively (Figure 5).

#### 3.1.3. Flexural Strength

**Figure 6:**
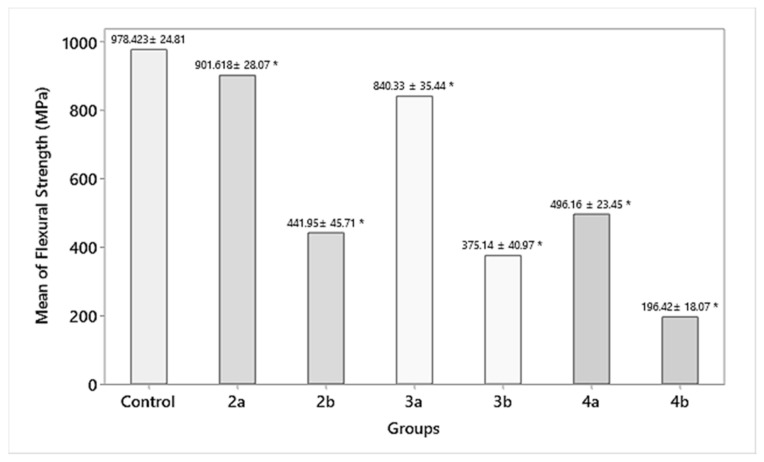
Flexural strength of the abraded zirconia samples (MPa). * Statistically significant difference between the groups.

While the control group (group 1) had a flexural strength of 978 ± 24.81 MPa, the specimens in the test groups—group 2a, 2b (901.618 ± 28.07, 441.95 ± 45.71 MPa); group 3a, 3b (840.33 ± 35.44, 375.14 ± 40.97 MPa); and group 4a, 4b (496.16 ± 23.45, 196.42 ± 18.07 MPa)—showed a statistically insignificant (*p* > 0.05) decrease in flexural strength for 1.5 mm (except group 4a, 4b, which showed a statistically significant (*p* = 0.000) decrease in flexural strength) and a significant decrease in flexural strength for 1 mm residual thickness, respectively (Figure 6). 

#### 3.1.4. X-ray Diffraction Analysis

**Figure 7:**
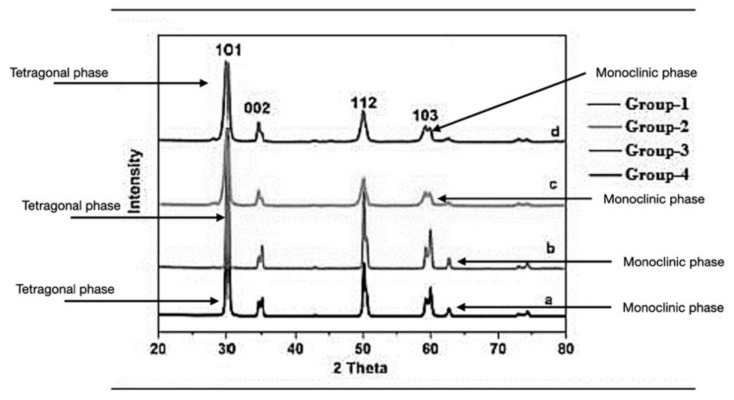
Patterns of X-ray diffraction of all tested groups.

The tetragonal crystalline phase for both of the residual thicknesses (1.5 and 1.0 mm) of monolithic zirconia was confirmed by X-ray diffraction pattern and peak analysis of the control group. After surface abrasion, the peak intensity of groups 2a and 2b, 3a and 3b, and 4a and 4b decreased. Compared with the control group, ground specimens presented an increase in FWHM (full width at half maximum) and asymmetrical broadening of the tetragonal peak. The lowest peak distortion was observed in group 4, as indicated in Figure 7. The grinding procedure had no significant effect on the relative amount of tetragonal zirconia in all tested groups. The tetragonal crystalline phase was confirmed. After abrasives, the peak intensity in the specimens of groups 2, 3, and 4 decreased, while the ground specimens presented asymmetrical broadening of the tetragonal peak and FWHM increase. Group 4 presented the least distortion, while grinding had no significant effect on tetragonal zirconia’s relative amount (Figure 7). 

Based on the individual results, diamond burs were selected as the best burs for chairside grinding protocols since the group showed the least change in the flexural strength as compared with all other burs.

### 3.2. Phase 2

In Phase 2, specimens were ground using diamond burs and were then tested on the same three physical and mechanical properties as mentioned before. The results were as follows:

#### 3.2.1. Surface Roughness

**Figure 8:**
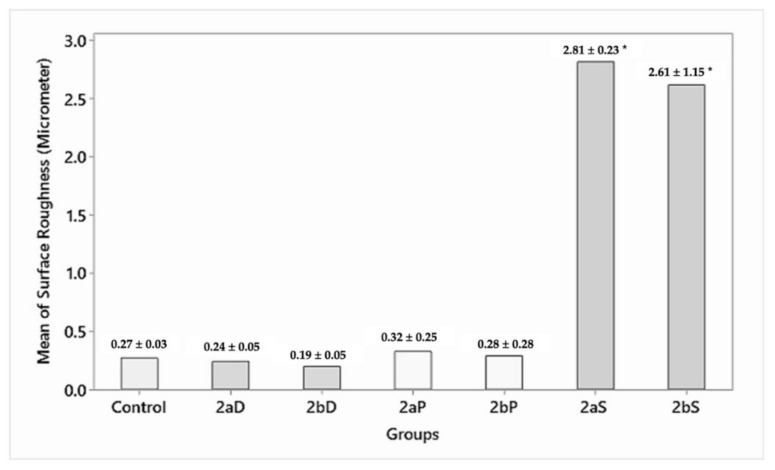
Surface roughness after polishing procedures (microns). * Annotates significant differences.

On the intragroup comparison, group D (0.24 ± 0.05, 0.19 ± 0.05 µm) showed a statistically significant (*p* = 0.000) decrease in surface roughness as compared with group P (0.32 ± 0.25, 0.28 ± 0.28 µm), which showed an insignificant (*p* > 0.05) increased surface roughness. On the other hand, group S (2.81 ± 0.23, 2.61 ± 1.15 µm) demonstrated a statistically significant (*p* < 0.05) increased surface roughness for specimens of 1.5 and 1 mm residual thicknesses, respectively. On the intergroup comparison, while the control group showed a surface roughness of 0.27 ± 0.03 µm, group 2aD, 2bD (0.24 ± 0.05, 0.19 ± 0.05 µm) showed a statistically significant decrease in surface roughness, and group 2aP, 2bP (0.32 ± 0.25, 0.28 ± 0.28 µm) showed a statistically insignificant increase (*p* > 0.05) in surface roughness. Whereas, group 2aS, 2bS (2.81 ± 0.23, 2.61 ± 1.15 µm) demonstrated a statistically significant increase in surface roughness for 1.5 and 1 mm residual thicknesses, respectively (Figure 8). 

#### 3.2.2. Surface Hardness

**Figure 9:**
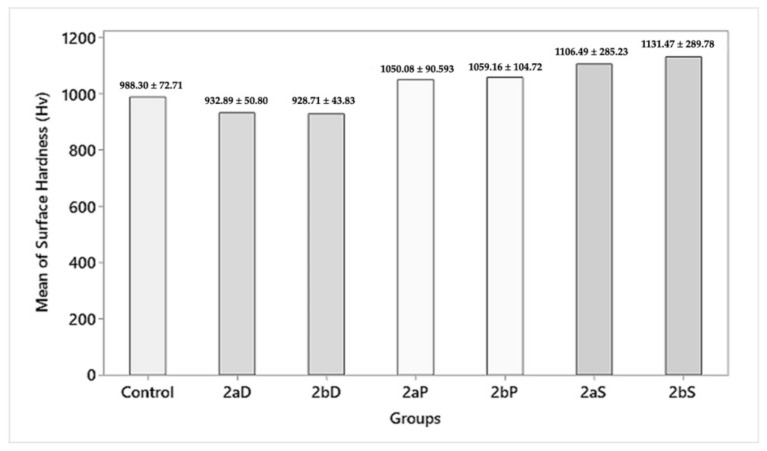
Surface hardness after polishing procedures (HV).

On the intragroup comparison, group 2aS, 2bS (1059.16 ± 104.72, 1131.47 ± 289.78 HV) showed a statistically insignificant (*p* > 0.05) increase in surface hardness as compared with group 2aD, 2aD (932.89 ± 50.80, 928.70 ± 43.83 HV) and group 2aP, 2bP (1050.08 ± 90.593, 1059.16 ± 104.72 HV) for 1.5 and 1 mm residual thicknesses, respectively. On the intergroup comparison, while the control group showed a surface hardness of 988.30 ± 72.71 HV, group 2aD, 2bD (932.89 ± 50.80, 928.70 ± 43.83 HV) showed a statistically insignificant (*p* > 0.05) decrease in surface hardness, and group 2aP, 2bP (1050.08 ± 90.593, 1059.16 ± 104.72 HV) and group 2aS, 2bS (1059.16 ± 104.72, 1131.47 ± 289.78 HV) showed statistically insignificant (*p* > 0.05) increases in surface hardness for 1.5 and 1 mm residual thicknesses, respectively (Figure 9).

#### 3.2.3. Flexural Strength

**Figure 10:**
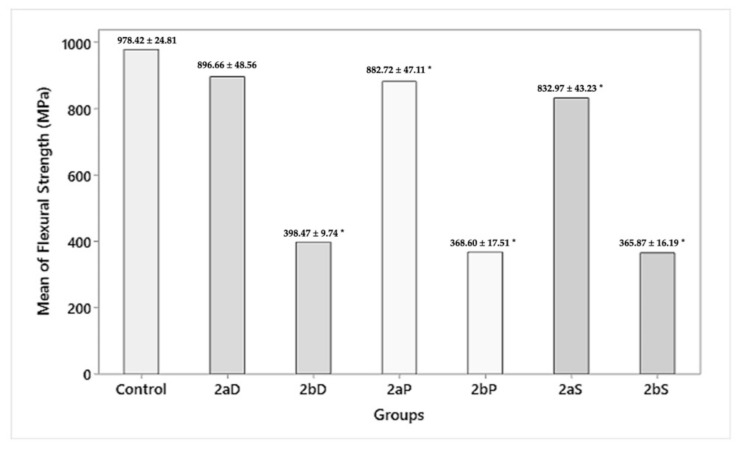
Flexural strength after polishing procedures (MPa). * Annotates significant differences.

On the intragroup comparison, there was a statistically insignificant (*p* > 0.05) difference between the flexural strengths of group 2aD (896.66 ± 48.56 MPa), group 2aP (882.72 ± 47.11 MPa), and group 2aS (832.97 ± 43.23 MPa) for 1.5 mm residual thickness. However, group 2bD (398.47 ± 9.74 MPa), group 2bP (368.60 ± 17.51 MPa), and group 2bS (365.87 ± 16.1 MPa 9) of 1 mm residual thickness showed a statistically significant (*p* = 0.000) difference in flexural strength when compared with 1.5 mm residual thickness. On the intergroup comparison, while the control group showed a flexural strength of 978.42 ± 24.81, group 2aD, 2bD (896.66 ± 48.56, 398.47 ± 9.74 MPa); group 2aP, 2bP (882.72 ± 47.11, 368.60 ± 17.51 MPa); group 2aS, 2bS (832.97 ± 43.23, 365.87 ± 16.19 MPa) showed a statistically insignificant (*p* > 0.05) decrease in flexural strength for 1.5 mm and a statistically significant (*p* = 0.000) decrease in flexural strength for 1 mm residual thickness, respectively (Figure 10).

**Figure 11:**
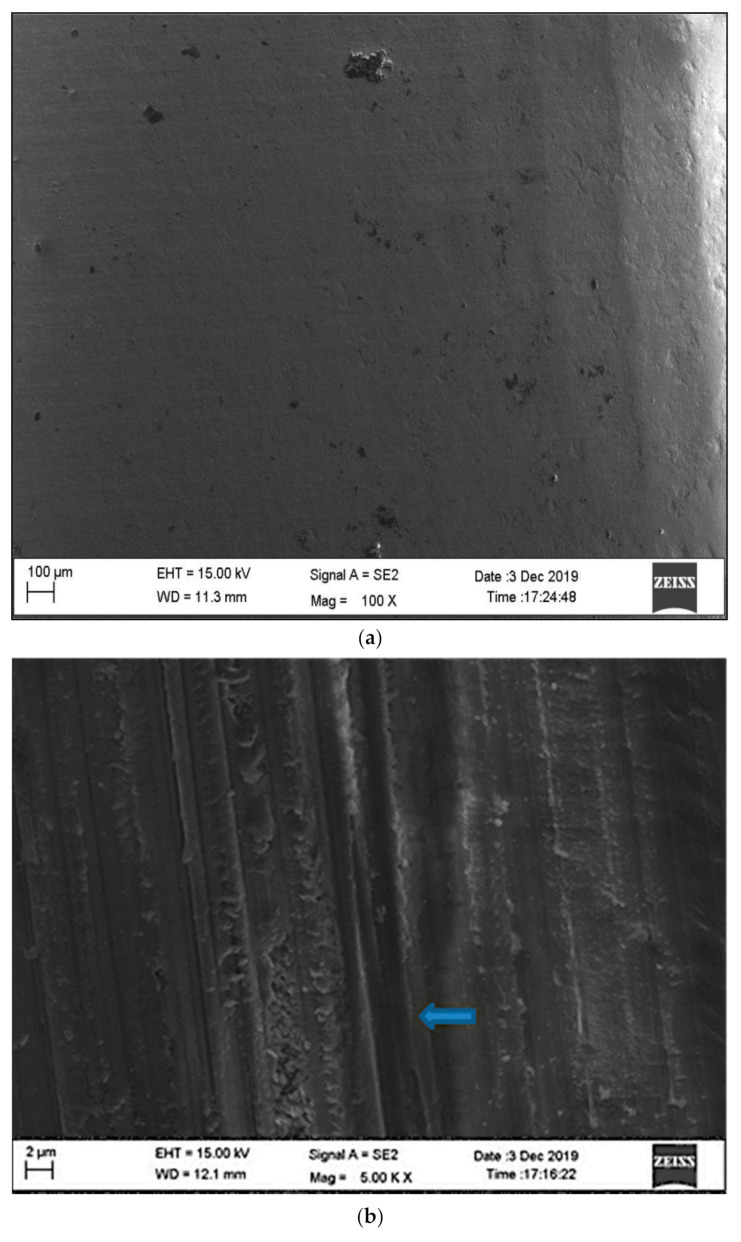

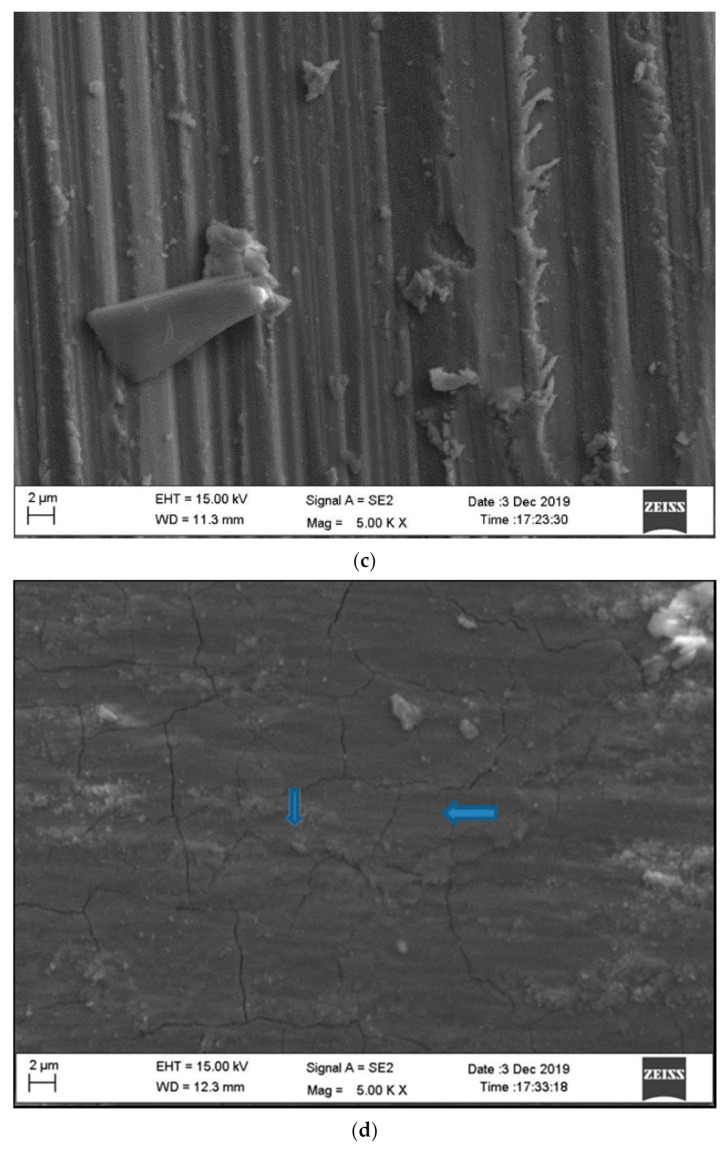
(**a**) SEM image of sintered zirconia surface (control group). (**b**) SEM image of zirconia surface abraded with diamond bur (notable striations with a nonhomogeneous surface). (**c**) SEM image of zirconia surface abraded with modified diamond bur (notable striation following direction of grinding). (**d**) SEM image of zirconia surface abraded with carbide bur (presence of surface cracks).

SEM images of sintered and abraded zirconia specimens are shown in Figure 11a–d.

## 4. Discussion

This study demonstrated that (1) the grinding and polishing agents employed were not able to re-establish smooth surfaces compared with the original surfaces. Therefore, the null hypothesis that different surface grinding and polishing agents would not affect the surface roughness, hardness, and flexural strength of monolithic zirconia was not supported by the findings of the present study. Furthermore, (2) the least change in roughness was noted in the zirconia specimens that were abraded with carbide burs, whereas the other two groups (abraded with diamond and modified diamond burs) showed a significant increase in the surface roughness values compared with the control group.

Chairside adjustment of monolithic zirconia is routinely performed by clinicians in order to eliminate occlusal interferences. The ultimate goal is to achieve a smooth surface similar to the glazed surface wherein the surface is well tolerated by the oral tissues and resists plaque accumulation [17]. For this reason, the restorations are either reglazed in the laboratory or polished chairside. Even though glazed restorations show smooth surfaces, the wear behavior is not superior in comparison with polished monolithic zirconia restorations [18]. Reglazed surfaces lead to greater antagonist wear than polished surfaces [19].

There could be several reasons to explain the above findings, such as induced strain causing a temperature rise, thereby leading to phase transformation. However, the ground specimens and their X-ray diffraction pattern disproved this assumption. The pattern showed reduction in the peaks in all the three groups, 2, 3, and 4, as compared with the peaks in the control group. A T→M phase transformation was not initiated, as the surface treatment was not effective enough. Broadening of the tetragonal peaks can be attributed to lattice distortion of the crystalline zirconia structure. 

In contrast to the above-mentioned studies, Ramos et al. [20] and Juy et al. [21] found out that a higher monoclinic phase can be promoted by grinding. The heat treatment of the test groups might have induced a reverse transformation of the monoclinic phase, resulting in a similar monoclinic phase content compared with the control group. 

Several studies (Ohkuma et al. [22], Güngör et al. [23], Hmaidouch et al. [24]) have concluded that the grit size of the coarse diamond particles used contributes to greater roughness values. In the present study, the high surface roughness in group 2 (specimens abraded with diamond bur) and group 3 (specimens abraded with zirconia diamond) is attributed to the grit size (100–125 µ/medium grit) of the diamond particles, leading to deeper surface flaws. Ra values for group 4 showed minimal surface roughness values and had a polishing effect, which might be attributed to the eight-bladed toothed geometry of the carbide bur, causing a polishing effect upon the zirconia.

The result of the surface hardness values obtained indicated an increase in the mean hardness of the zirconia specimens in all the three groups compared with the control group. The increase in hardness between these groups tested was statistically insignificant. Groups 2, 3, and 4 demonstrated hardnesses of 1069 HV, 1056 HV, and 1081 HV, respectively. The increase in hardness in the test groups could be attributed to the fact that grinding can induce the strained cubic phase, but it is not strong enough to trigger transformation toughening (t-m or t-c). This is in agreement with studies by Pittayachawan et al. [25] and Traini et al. [26]. The reported differences between these were statistically insignificant.

A significant outcome of this study was the detrimental effect that the tungsten carbide burs had on the mean flexural strength of the dental zirconia specimens. The mean flexural strength was reduced by more than half (496.16 ± 23.45 and 196.42 ± 18.07 for 1.5 and 1 mm residual thicknesses, respectively) with the tungsten carbide finishing bur when compared with the control group (978 ± 24.81). The cutting mechanism of tungsten carbide burs was found to be the contributing factor for the reduction of the flexural strength. This is in agreement with the results of a study conducted by Botelho et al. [12], where sheetlike areas were observed in the specimens after grinding with tungsten carbide under SEM. There have been conflicting reports in the literature regarding the use of tungsten carbide burs. Ferrari and Conti [27] concluded that tungsten carbides have a better finishing potential as compared with diamond points. Similarly, Ercoli et al. [28] in their study demonstrated the superior performance of carbides in comparison with diamond points. However, Wang et al. [29] concluded that tungsten carbides are not recommended to grind zirconia as they lead to reduced biaxial flexural strength. In order to comprehensively understand the effect of carbides on the roughness and strength of zirconia, they were included as the comparison group in this study. For materials such as zirconia, the surface damage facilitating premature crack propagation under loading and localized heat development is responsible for the strength degradation. However, in the present study, the scanning electron microscopy images of the zirconia specimens abraded with diamond burs (groups 2 and 3) produced notable striations with nonhomogeneous surfaces. Specimens ground using tungsten carbide burs showed flat smooth surfaces and cracks in the grinding direction. Higher magnification revealed further smaller cracks tangential to the grinding direction. Although carbide bur treatments resulted in smooth zirconia finish, their use is not recommended, as it has a negative impact on the flexural strength. Previous authors have advocated for the use of tungsten carbide burs provided it is followed by air-particle abrasion [30].

While the effect of diamond abrasion in groups 2 and 3 had a reduction in the mean flexural strength when compared with the control group, this reduction in strength was not statistically significant. This result supports the notion that zirconia strength may not be affected by surface roughness.

In the current study, there was no relationship between the mean surface roughness and the mean flexural strength. The diamond burs produced a significant difference in the mean surface roughness (control), and conversely, a minimal difference was observed in the mean surface roughness of the tungsten carbide bur group, yet they did feature a significantly reduced mean flexural strength compared with the control. Finishing with diamond burs is the preferred finishing protocol since the reduction in flexural strength is less compared with the carbide group.

After grinding the specimens with diamond burs, the specimens were then subjected to various chairside polishing protocols. These polished specimens were then tested on the same three properties, vis-à-vis surface roughness, surface hardness, and flexural strength. Alumina and diamond are commonly used as the abrasive particles in commercial polishing pastes. Zirconia is also used as an abrasive in the industry and is found in C-type silicone points (Shofu Dental, Kyoto, Japan), which are used to polish composite resin [31]. Porcelain polishing systems were included in this study as these kits are routinely used. Zirconia polishing kits are newer entities. Although zirconia restorations are commonly used by clinicians, the adoption of polishing kits in daily practice is limited.

The study also investigated the effectiveness of the novel zirconia slurry used for polishing. The results indicated that the zirconia slurry could not create a smooth surface and generated a significantly higher Ra value when compared with the control group (group 1) (Figure 11). However, this group did not show any significant changes in the mean hardness and flexural strength values. 

Polishing with diamond paste and a polishing kit generated smooth surfaces, indicating that polishing can remove defects induced by adjustments having a more favorable distribution, reducing surface roughness in spite of induction of further defects [20]. 

The study evaluated the flexural strength after polishing the abraded samples, and a reduction in the flexural strength was observed in all the groups, although this was statistically insignificant. This is in agreement with the studies performed in the literature [9,26]. This could be attributed to the fact that the polishing process relieves the strain and compressive stresses within the surface of specimens without transforming the tetragonal phase to the monoclinic phase.

Studies comparing different abrasive agents and grinding of zirconia at varied amounts in order to determine the best method of abrading and also the critical thickness of the restorative material should be conducted in the future. More clinical studies should be conducted to test the longevity of repolished zirconia FPDs.

## 5. Conclusions

The following conclusions could be drawn from this study:

From a clinical standpoint, chairside occlusal adjustments of monolithic zirconia restorations should be avoided, as they could lead to impairment of its mechanical properties. However, if these adjustments are needed in order to attain occlusal harmony, diamond burs of standard- or fine-grit-size particles should be considered. Additionally, the ground surface should be chairside polished with either a polishing kit or diamond paste. Grinding with a diamond bur followed by polishing instead of reglazing helps retain the mechanical properties of the monolithic ceramic material. 

## Figures and Tables

**Table 1:** Characteristics of grinding agents tested.

Type of Grinding Agents	Commercial Names	Design and Features
Diamond points	Mani, Japan	100–125 µ/medium grit, cylindrical
Modified diamond points for zirconia	Predator Zirconia Diamonds, Prima Dental, India	110–130 µ/medium grit, cylindrical
Tungsten carbide burs	Predator 2 Turbo, Prima Dental, India	Medium-sized, straight

**Table 2:** Characteristics of polishing agents tested.

Types of Polishing Agents	Commercial Names
Diamond polishing paste	Diamond polishing paste (Signum HP diamond, Kulzer, Germany), size: 3 um, polishing wheel (Z-Shine, Dental Creations Ltd., Waco, TX, USA)
Porcelain-specific polishing paste	Shofu Dental Corporation, San Marcos, TX, USA (size 2–4 um)
Novel zirconia slurry	Indigenously manufactured (size: 2–4 um)

## Data Availability

No data reported.
